# Identification of a dual TAOK1 and MAP4K5 inhibitor using a structure-based virtual screening approach

**DOI:** 10.1080/14756366.2020.1843452

**Published:** 2020-11-09

**Authors:** Min-Wu Chao, Tony Eight Lin, Wei-Chun HuangFu, Chao-Di Chang, Huang-Ju Tu, Liang-Chieh Chen, Shih-Chung Yen, Tzu-Ying Sung, Wei-Jan Huang, Chia-Ron Yang, Shiow-Lin Pan, Kai-Cheng Hsu

**Affiliations:** a Graduate Institute of Cancer Biology and Drug Discovery, College of Medical Science and Technology, Taipei Medical University, Taipei, Taiwan; b School of Pharmacy, College of Medicine, National Taiwan University, Taipei, Taiwan; c Master Program for Cancer Molecular Biology and Drug Discovery, College of Medical Science and Technology, Taipei Medical University, Taipei, Taiwan; d Ph.D. Program for Cancer Molecular Biology and Drug Discovery, College of Medical Science and Technology, Taipei Medical University, Taipei, Taiwan; e Ph.D. Program in Biotechnology Research and Development, College of Pharmacy, Taipei Medical University, Taipei, Taiwan; f TMU Research Center of Cancer Translational Medicine, Taipei Medical University, Taipei, Taiwan;; g Warshel Institute for Computational Biology, The Chinese University of Hong Kong, Shenzhen, P. R. China; h School of Life and Health Sciences, The Chinese University of Hong Kong, Shenzhen, P. R. China; i Institute of Bioinformatics and Systems Biology, National Chiao Tung University, Hsinchu, Taiwan; j School of Pharmacy, Taipei Medical University, Taipei, Taiwan; k Graduate Institute of Pharmacognosy, Taipei Medical University, Taipei, Taiwan; l Biomedical Commercialization Center, Taipei Medical University, Taipei, Taiwan; m Cancer Center, Wan Fang Hospital, Taipei Medical University, Taipei, Taiwan

**Keywords:** Structure-based virtual screening, STE20 pathway, small-molecule, kinase inhibitor, cancer, drug discovery

## Abstract

The STE20 kinase family is a complex signalling cascade that regulates cytoskeletal organisation and modulates the stress response. This signalling cascade includes various kinase mediators, such as TAOK1 and MAP4K5. The dysregulation of the STE20 kinase pathway is linked with cancer malignancy. A small-molecule inhibitor targeting the STE20 kinase pathway has therapeutic potential. In this study, a structure-based virtual screening (SBVS) approach was used to identify potential dual TAOK1 and MAP4K5 inhibitors. Enzymatic assays confirmed three potential dual inhibitors (>50% inhibition) from our virtual screening, and analysis of the TAOK1 and MAP4K5 binding sites indicated common interactions for dual inhibition. Compound 1 revealed potent inhibition of colorectal and lung cancer cell lines. Furthermore, compound 1 arrested cancer cells in the G0/G1 phase, which suggests the induction of apoptosis. Altogether, we show that the STE20 signalling mediators TAOK1 and MAP4K5 are promising targets for drug research.

## Introduction

Kinases are important signalling mediators that maintain cellular homeostasis. They are tightly regulated and can control various functions, such as cell proliferation, apoptosis and differentiation^1^. Because of their wide use as signalling mediators, aberrations to kinase signalling can lead to a variety of human maladies, such as immune, neurological or cancer disorders^1–3^. As a result, kinases are therapeutic targets of interest.

There has been great success with small-molecule kinase inhibitors. Imatinib, the first FDA approved kinase inhibitor, has shown success as a therapeutic drug and has paved the way for more kinase targeting small-molecule inhibitors^4^. Currently, the FDA has approved a total of 52 small-molecule kinase inhibitors^5^. However, many of the inhibitors were designed with a single target. Complex diseases, such as cancer, can have multiple pathological manifestations^6^. An inhibitor with only one protein target may reduce growth, but the proliferation of the malignant cell can be reactivated through other signalling pathways^6–8^. In addition, cancer cells have been known to develop resistance to small-molecule inhibitors through mutations to their targeted kinase binding site^7^. A multi-target kinase inhibitor may circumvent ineffectiveness due to mutations in a single targeted binding site^9^. Dual kinase inhibitors, such as Dasatinib and Lapatinib, have shown increased efficacy for inhibiting SRC/ABL and EGFR/HER2, respectively^10^^,^^11^. A multi-targeting kinase inhibitor provides a beneficial therapeutic strategy by inhibiting multiple aberrant kinases in signalling pathways.

The STE20 (Sterile 20) protein kinase family consists of a total of 30 serine-threonine kinases that can be grouped into 10 subfamilies^12^. This kinase family has been implicated in the regulation of cytoskeletal rearrangements and actin stress fibre disassembly^13^ and modulation of the MAPK cascade stress responses can have an impact on cellular processes, such as proliferation, cell cycle, apoptosis and differentiation^14^^,^^15^. STE20 kinases have been reported to have roles in cancer cell proliferation, transformation, motility and invasion^16^^,^^17^. STE20 kinase inhibitors have been designed for potential cancer treatment^17–19^. However, more research is needed to identify potential therapeutic compounds.

Both MAP4K5 and TAOK (Thousand-and-one amino acid kinase) belong to the mammalian STE20 kinase family^19^^,^^20^. MAP4K5, also named kinase homologous to STE20 (KHS1) or germinal centre kinase related (GCKR), phosphorylates various downstream kinase cascades, such as MKK4/MKK7, c-Jun N-terminal kinase (JNK) and stress-activated protein kinase (SAPK) cascades^21^. TAOK consists of a family of three kinases (TAOK1, TAOK2 and TAOK3). TAOK1 can promote structural changes to a cell by activating the MAPK cascade^19^. Both MAP4K5 and TAOK1 are involved with the MAPK signalling, which is a complex signalling cascade linked to organ development and tissue homeostasis^22^^,^^23^.

Irregular expression of these two kinases have been linked to various diseases. MAP4Ks were reported to modulate tumour cell transformation, invasion, and adhesion^20^^,^^24^. Overexpression of MAP4K5 has been linked to acute myeloid leukaemia^25^ and MAP4K5 has prognostic and functional significance in cancer. A recent study has also suggested that inhibition of MAP4K5 is correlated strongly with motor neuron survival^26^. Research has shown that inhibition of TAOK1 can induce mitotic cell death in breast cancer cells and TAOK1 dysregulation has also been linked to neurodegeneration in humans^19^^,^^27^^,^^28^. This suggests that MAP4K5 and TAOK1 are viable targets for therapy. In addition, they are involved in complex disease with a complex signalling pathway. Targeting multiple kinases within a signalling cascade may overcome resistance observed with single target kinase inhibitors, such as mutations with the ATP binding site^7^. Therefore, a small-molecule targeting both TAOK1 and MAP4K5 has great potential to elucidate our understanding of their involvement in disease as well as having potential for great therapeutic benefits.

In this study, we employed a structure-based virtual screening (SBVS) to identify potential inhibitors targeting TAOK1 and MAP4K5. The SBVS is a cost-efficient process that can lead to compound discovery and optimisation. In short, compounds from a library were docked into the structure of the target protein binding site. Potential inhibitors were selected based on the docking scores ^29^. To identify dual TAOK1 and MAP4K5 inhibitor, the docking scores for each target kinase were combined to produce a consensus score. We selected the top-ranked compounds based on the consensus score and tested them using an enzyme-based assay against TAOK1 and MAP4K5. The compounds were validated for inhibitory activity. The compounds produced common interactions in the binding sites of TAOK1 and MAP4K5. Validated compounds were further tested by *in vivo* assays that suggest apoptosis was induced in cancer cells when treated with the compounds. Together, this suggests that the identified compounds are dual-targeting kinase inhibitors and have therapeutic potential.

## Material and method

### Preparation of compound library

The NCI compound database (roughly 260,000 compounds) was selected for screening. The library was first filtered using a HTS filter in Pipeline Pilot^30^, which filters molecules that are likely to be poor candidates for high-throughput screening. Conditions for removal include compounds with non-organic atom types, reactive substructures, and compound with a molecular weight > 150. This resulted in 240,000 compounds. A pan assay interference compounds (PAINS) to reduce false-positives was then applied^31^. The PAINS substructures were obtained from the ZBH – Centre of Bioinformatics, Hamburg University. Roughly 210,000 compounds remained. Next, compounds without drug-like features were filtered. This process includes the “Lipinski and Verber Rules Filter” component in Pipeline Pilot^30^ and left roughly 190,000 compounds. Compounds with a heterocyclic ring system can form hydrogen-bond interactions with hinge residues ^32^. Therefore, compounds without a heterocyclic ring system were filtered, leaving 120,000 compounds. Finally, the remaining compounds were docked into MAP4K5 and TAOK1 using the molecular docking software LeadIT^33^.

### Virtual screening for potential dual inhibitors

Virtual screening of the NCI compound database was performed using the molecular docking software LeadIT^33^. At the time of this study, a crystal structure for both MAP4K5 and TAOK1 was unavailable. As a result, a homology model was produced using the MODELLER^34^ function in the Chimaera^35^ software. The protein structures MAP4K3 (PDB ID: 5J5T) and TAOK2 (PDB ID: 2GCD) were used to create the homology model for MAP4K5 and TAOK1, respectively. The structures were obtained from the Protein Data Bank^36^. A space of 10 Å from the template co-crystal ligand was determined as the binding site. Within the ligand-binding option, the software presents three options: a hybrid approach (enthalpy and entropy), enthalpy (classic triangle matching) and entropy (single interaction scan). Here, we used the default setting (i.e., a hybrid approach) for the docking analysis. The solutions per iteration and fragmentation were both set to a maximum of 200. All other docking parameters used default settings. A consensus score (*CS*) for each compound was generated. The *CS* score represents compound potential as a dual MAP4K5 and TAOK1 inhibitor. Here, the *CS* of the compound *c* is defined as

$$CS(c)=R_{MAP4K5}(c)+R_{TAOK1}(c)$$

where *R_MAP4K5_*(*c*) and *R_TAOK1_* (*c*) are the ranking score of compound *c* in MAP4K5 and TAOK1, respectively. Compounds were then filtered based on the presence of a hydrogen bond with hinge residues^37^. The 100 top-ranked compounds were selected as potential dual inhibitors. In total, seven compounds were requested from the NCI database based on availability.

### Enzymatic assay

The enzymatic assay was outsourced to Thermo Fisher Scientific to conduct. They used LanthaScreen and Z´-LYTE technology, which were both robust homogeneous assays method by Fluorescence Resonance Energy Transfer (FRET) to evaluate TAOK1 and MAP4K5, respectively. Briefly, the test compound was co-incubated with fluorescein-labelled substrate, kinase, ATP, kinase buffer and development reagent for reaction. After 1 h, the stop solution (EDTA) was added and then the results were determined by a fluorescence reader. The data was shown as an average of two replicates.

The kinase selectivity of selected compounds was performed by Thermo Fisher Select Screen service. A kinome screen occurred across a panel of roughly 70 kinases. Conditions and analyses are available online (www.thermofisher.com/selectscreen).

### Cell culture

Human colorectal cancer cell lines HCT116, HT-29 and human lung cancer cell line, H1299 were purchased from the Bioresource Collection and Research Centre (BCRC, Taiwan) and were incubated either in McCoy’s 5 A medium or RPMI-1640 medium (Thermo Fisher Scientific, Walthma, MA, USA), supplemented with 10% foetal bovine serum and 1% Penicillin-Streptomycin-Amphotericin B Solution (Biological Industries, Kibbutz Beit-Haemek, Israel) at 37 °C in a humidified atmosphere with 5% CO_2_.

### Cell viability

Cell viability was determined by MTT (3–(4,5-dimethylthiazol-2-yl)-2,5-diphenyltetrazolium bromide, Sigma Chemical Co, St. Louis, MO, USA) assay. Cells were seeded in 96-well plates at a density of 5,000 cells/well in a regular culture medium overnight. Then, the cells were treated with various concentrations of indicated compounds for 72 h. After that, the medium was removed, 100 µL MTT solution (0.5 mg/ml in phosphate-buffered saline, PBS) was added to each well and then incubated for 1 h at 37 °C. The insoluble formazan dyes, representing the active mitochondrial dehydrogenase in live cells, were dissolved in the dimethyl sulfoxide (DMSO). The absorbance was analysed by an ELISA reader (Beckman Coulter, Brea, CA, USA) at 550 nm.

### Cell proliferation

SRB (sulforhodamine B, Sigma Chemical Co, St Louis, MO, USA) assay was used to analyse cell proliferation. Cells were seeded in 96-well plates at a density of 5000 cells/well and then treated with various concentrations of indicated compounds for 48 h. Cells were fixed with 10% TCA (trichloroacetic acid), washed with PBS, and stained with 100 µL SRB solution (0.4% SRB in 1% acetic acid) for 15 min. Then, cells were washed three times with 1% acetic acid and dissolved in the trizma base solution (10 mM). The absorbance was measured by an ELISA reader (Beckman Coulter Diagnostics, Brea, CA, USA) at 510 nM. 

### Cell cycle distribution

The cell cycle distribution was measured by flow cytometry. HT-29 cells were treated with or without the indicated concentrations of compound 1 for 24 or 48 h or 72 h. The cells were harvested by trypsinizing and fixed in ice-cold 75% ethanol for 1 h at −20 °C. The samples were washed with PBS and resuspended in the proper amount of DNA extraction buffer, containing 0.2 M Na_2_HPO_4_ and 0.1 M citric acid pH 7.8, for 30 min. Then the cells were washed again with PBS and stained with propidium iodide solution containing 100 µg/ml RNaseA, 80 µg/ml propidium iodide and 0.1% Triton X-100, which were all in PBS. Finally, cell cycle distribution was determined and performed by utilising BD Accuri™ and C6 Software (BD Biosciences, Franklin Lakes, NJ, USA).

### Compound structure comparison

The structure of each compound was represented by an atom-pair fingerprint generated by the RDKit Fingerprint tool in KNIME^30^. A similarity matrix was created using the Pearson Correlation Coefficient to produce a similarity score. A similarity score of 0 denotes little similarity, while a score of 1 denotes high similarity. The software tool Morpheus (https://software.broadinstitute.org/morpheus/) was used to generate the heatmap.

### Chemical purity

The purities of compounds were determined by HPLC. The HPLC system was using a C-18 column (150 × 4.6 mm, Ascentis) by an L-2130 pump (Hitachi, Ibaraki, Japan) and a UV/vis L-2420 detector (Hitachi, Ibaraki, Japan).

## Result

### Overview of identifying potential dual inhibitors

**Figure 1.**
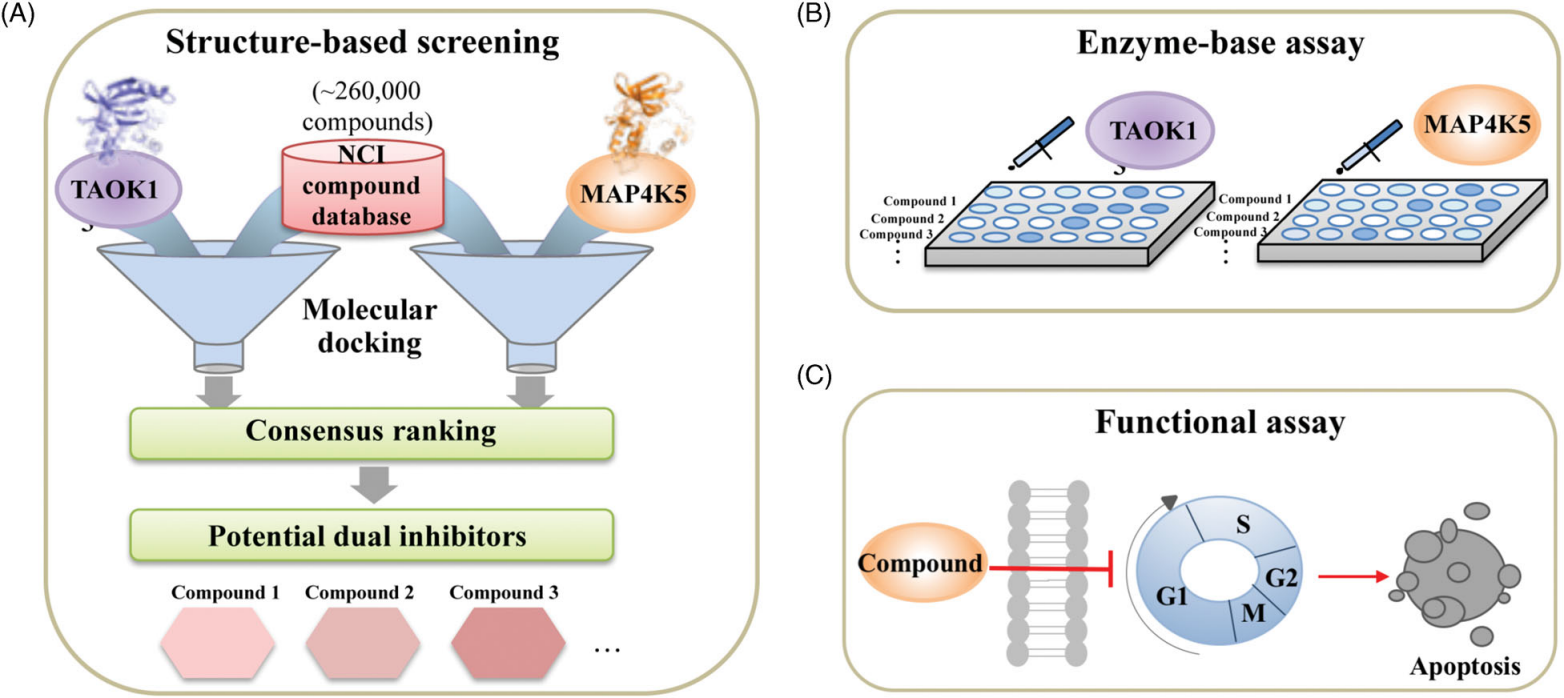
Overview of study. (A) A compound library was virtually screened to identify potential inhibitors targeting TAOK1 and MAP4K5. (B) Top-ranked potential inhibitors are selected for enzyme-based assays. (C) Potential inhibitors are further tested using functional assays to monitor *in vitro* cell death.

We sought to identify novel dual inhibitors targeting TAOK1 and MAP4K5 of the STE20 kinase family. To identify novel dual inhibitors, we screened the NCI compound database (roughly 260,000 compounds) using a SBVS approach (Figure 1(A)). Structural information of a protein target can be used to discover important ligand-protein interactions to identify new inhibitors quickly^38^. Because the crystal structure for both TAOK1 and MAP4K5 is unavailable at the time of this study, we created a homology model based on their closest relatives, TAOK2 and MAP4K3, respectively. Compounds were docked into the binding site of each target and a consensus score was created for each compound. Finally, the compounds were ranked based on their consensus score and the top-ranked compounds were selected for validation (Figure 1(B)). Identified inhibitors were further validated using cell-based assays (Figure 1(C)).

### Selection and validation of dual inhibitors

**Figure 2.**
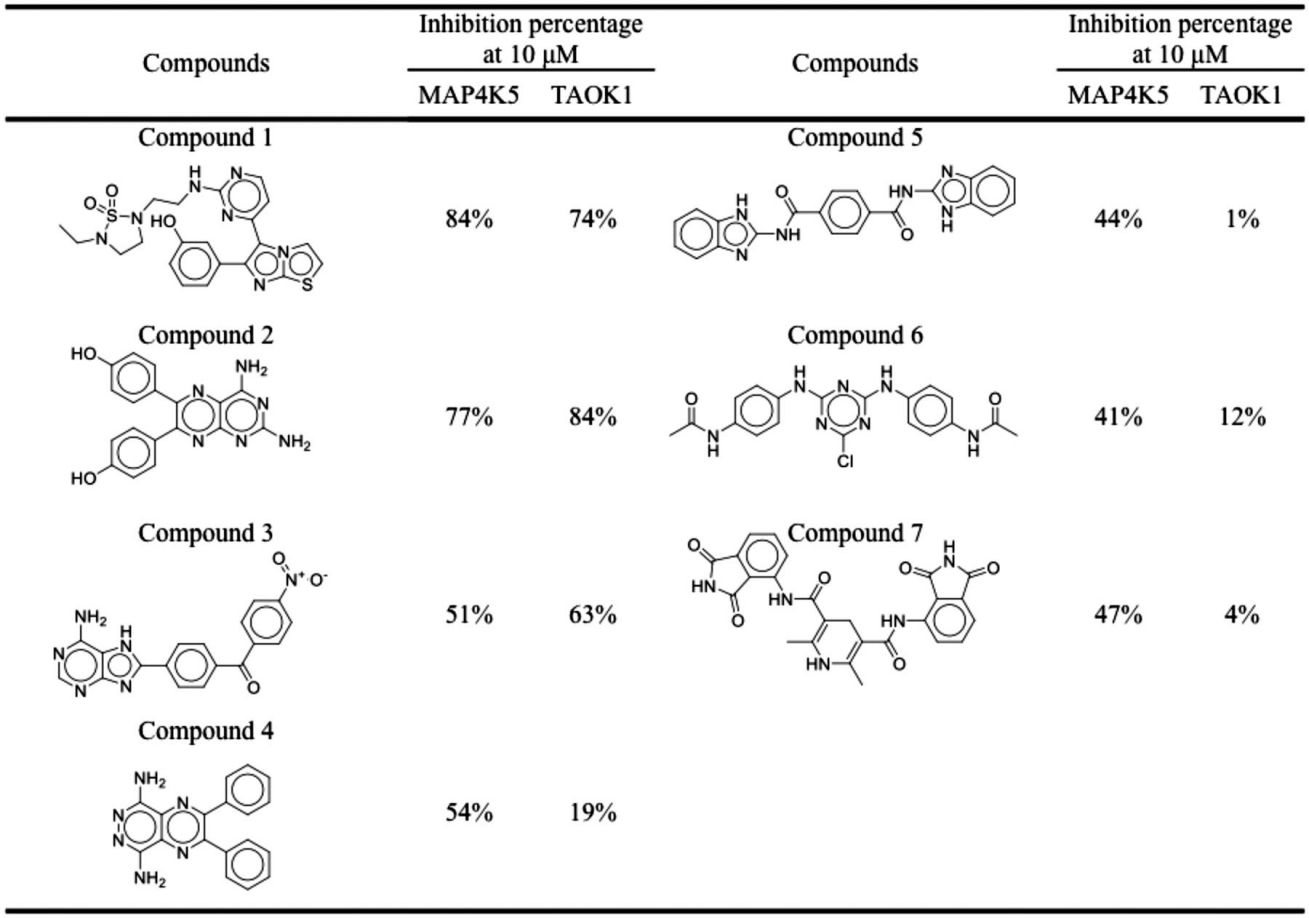
Structures and inhibitory percentages of potential inhibitors. A selection of seven compounds and their inhibitory activity towards MAP4K5 and TAOK1.

In absence of a three-dimensional structure of MAP4K5 and TAOK1, a homology model was used to predict the structure for both target kinases for virtual screening. The target protein sequences were subjected to a BLAST query in the Chimaera software^35^. The closest structure for MAP4K5 (PDB ID: 5J5T) was MAP4K3, which had an alignment score of 480 with 84.88% identity. The closest structure for TAOK1 was found to be TAOK2 (PDB ID: 2GCD), with an alignment score of 492 and an identity of 89.37%. Compounds were then docked into the ATP-binding site of modelled structure using LeadIT^33^. The ATP-binding site of the kinase superfamily is highly conserved and many kinase inhibitors targeting the ATP-binding site form hydrogen bonds to hinge residues^37^^,^^39^. The hinge consists of residues E76, Y77, and C78 for MAP4K5 and residues E79, Y80 and C81 for TAOK1. Compounds that did not form a hydrogen bond with at least one of the hinge residues were removed. A set of top-ranked compounds (3,000 compounds) were created for each protein target based on their docking score. Next, a consensus score (*CS*) for each compound was created. A compound with a high *CS* score has greater potential as a dual MAP4K5 and TAOK1 inhibitor. Upon ranking the docked compounds, a total of seven compounds were selected for enzyme-based assays. Each compound was tested at a dose of 10 µM against MAP4K5 and TAOK1 (Figure 2). Three compounds, 1, 2 and 3, produced an inhibition percentage of ≥50% against the two kinase targets. Purities of the compounds were determined using HPLC. All compounds had a purity of at least 92% (Supplemental Figure 1). These compounds were selected for further testing to identify their IC_50_ values. All three compounds showed favourable IC_50_ values, with compound 2 showing the most favourable value of 2.00 and 1.83 µM against MAP4K5 and TAOK1, respectively (Table 1 & Supplemental Figure 2). Therefore, our screening protocol identified three dual MAP4K5 and TAOK1 kinase inhibitors.

**Table 1.** The activity of the compounds against MAP4K5 and TAOK1.

Compound	MAP4K5 IC_50_ (μM)	TAOK1 IC_50_ (μM)
Compound 1	2.25	4.15
Compound 2	2.00	1.83
Compound 3	6.28	5.30

### Interaction analysis of inhibitors and MAP4K5

**Figure 3.**
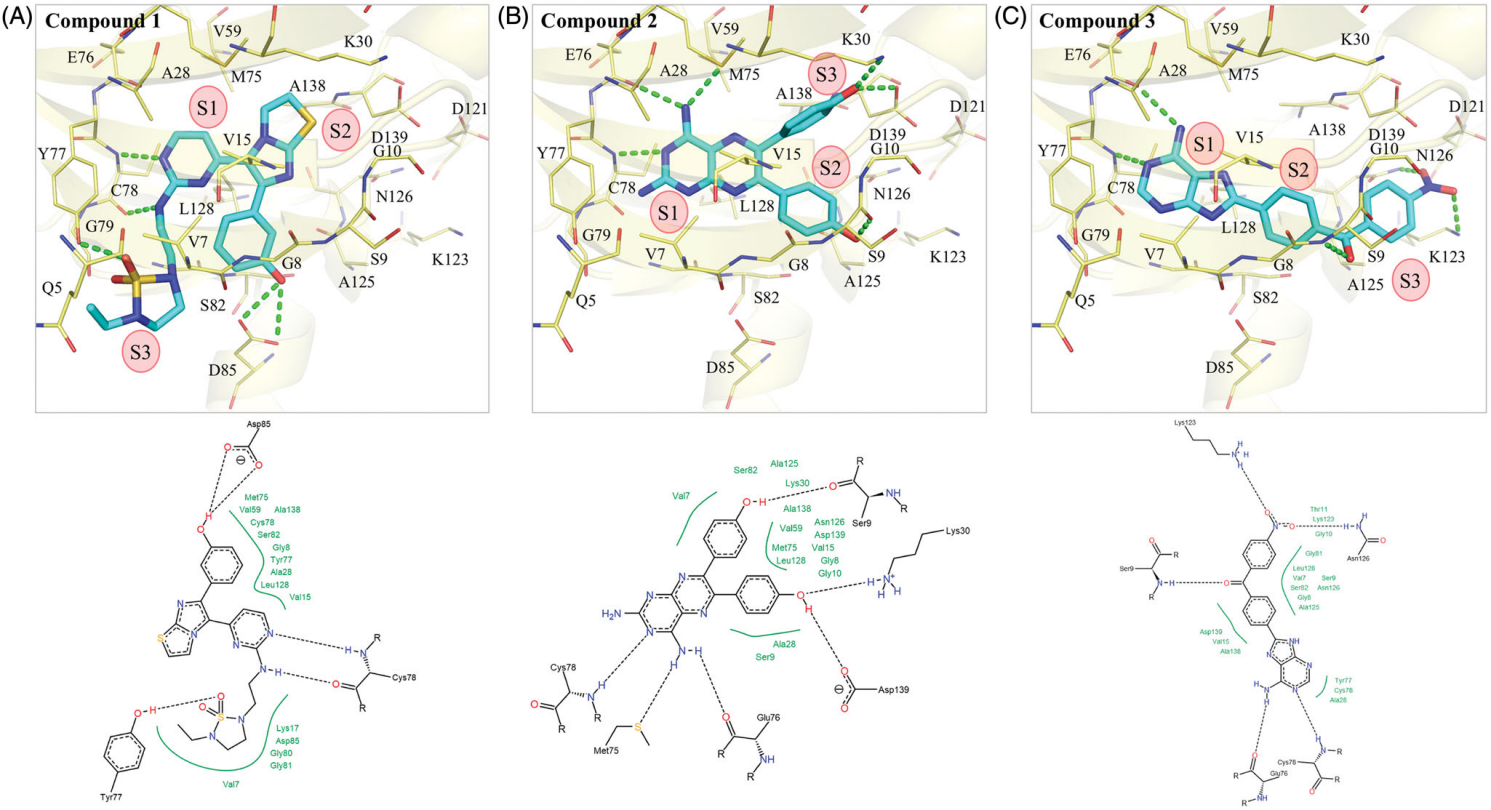
Interaction analysis of compounds in MAP4K5. The poses of (A) compound 1, (B) 2, and (C) 3 in MAP4K5. Compounds are coloured blue. MAP4K5 is coloured yellow. Residues are labelled as shown. Dashed green lines represent hydrogen bonds.

To determine the molecular interactions between the identified inhibitors and MAP4K5, we performed an interaction analysis using the molecular software LeadIT^33^. For clarity, the compounds are separated into three sub-structures. The S1 sub-structure (2-aminopyrimidine) of compound 1 forms hydrogen bonds with the nitrogen and oxygen of MAP4K5 hinge residue C78. Formation of hydrogen bonds with hinge residues is typical for small-molecule kinase inhibitors targeting the binding site ^37^. The cyclic nitrogen acts as a hydrogen acceptor, while the amine acts as a hydrogen donor for the nitrogen and oxygen backbone of C78, respectively (Figure 3(A)). The sub-structures S2 and S3, which form the terminal ends of compound 1, also form hydrogen bonds (Figure 3(A)). Two hydrogen bonds are formed betweenresidue D85 and the phenol of the sub-structure S2. The phenol of sub-structure S2 is attached to an imidazo[2,1-b][1,3]thiazole. Residue Y77 forms a hydrogen bond with the 1,2,5-thiadiazolidine 1,1-dioxide of the S3 sub-structure (Figure 3(A)). Hydrophobic interactions also occur within the MAP4K5 binding site. For example, residue A128 forms hydrophobic interactions with the S1 sub-structure. The S2 sub-structure creates hydrophobic contacts with residues V7 and A138, while the S3 sub-structure is sandwiched by hydrophobic interactions with residues V7, V15, L128 (Figure 3(A)).

Compound 2 forms hydrogen bonds with residues M75, E76 and C78 in MAP4K5 with its S1 sub-structure, pteridine-2,4-diamine (Figure 3(B)). The hinge residue C78 forms a hydrogen bond with the cyclic nitrogen. Residues M75 and E76 form hydrogen bonds with the amino group, which acts as a hydrogen donor for the two residues. Hydrophobic interactions by residues V15, A28, Y77, C78 and A128 sandwich the S1 sub-structure (Figure 3(B)). Attached to the sub-structure S1 are two phenol moieties that form sub-structures S2 and S3 (Figure 3(B)). One phenol group forms a hydrogen bond with residue S9, while the other creates a hydrogen bond with residues K30 and D139. Phenols consist of hydrophobic benzenoid rings. As a result, the two phenol moieties also form hydrophobic interactions with a number of residues, such as V15, L128, and A138 (Figure 3(B)).

 The S1 sub-structure of compound 3 contains a purin-6-amine moiety that forms interactions with hinge residues E76 and C78 (Figure 3(C)). The purin-6-amine is sandwiched by hydrophobic interactions with residues V7, A28, Y77 and C78 (Figure 3(C)). The S2 sub-structure consists of a benzaldehyde that links the S1 sub-structure and the S3 (nitrobenzene) sub-structure. The S2 sub-structure spans a hydrophobic channel created by residues V15 and K128 within the MAP4K5 active site. The benzaldehyde linker forms a hydrogen bond with residue S9 (Figure 3(C)). The S3 sub-structure contains a nitrobenzene, which forms hydrogen bonds with residues K123 and N126. The S3 sub-structure in MAP4K5 occupies a hydrophobic pocket that consists of residues, such as V15, A125 and L128 (Figure 3(C)). Together, the interaction analysis shows that the compounds target the MAP4K5 binding site.

### Interaction analysis of inhibitors and TAOK1

**Figure 4.**
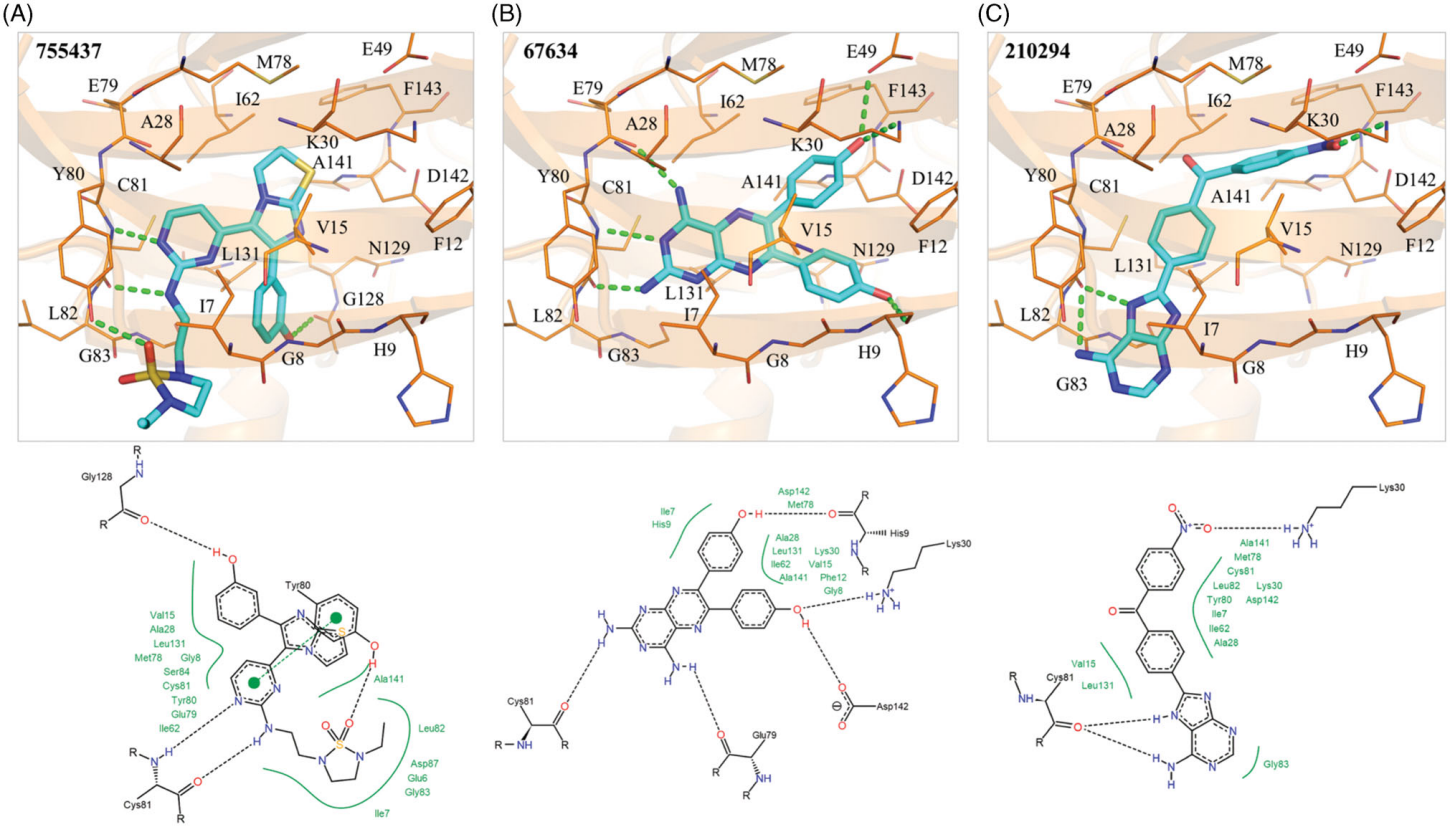
Interaction analysis of compounds in TAOK1. Docked poses of (A) compounds 1, (B) 2, and (C) 3 in TAOK1. Compounds are coloured blue, while TAOK1 is in orange. Dashed green lines represent hydrogen bonds. Residues are labelled as shown.

The enzymatic assays suggested that the three identified compounds in this study have TAOK1 inhibitory activity. An interaction analysis revealed molecular interactions between the compounds and TAOK1. The S1 sub-structure of compound 1 forms two hydrogen bonds with hinge residue C81 (Figure 4(A)). The cyclic nitrogen acts as a hydrogen acceptor, while the amine acts as a hydrogen donor for the nitrogen and oxygen backbone of C81, respectively (Figure 4(A)). The S2 sub-structure forms a hydrogen bond by accepting hydrogen from residue Y80 (Figure 4(A)). On the opposite end of compound 1, S3 contains a phenol group that forms a hydrogen bond with the backbone of residue G128 (Figure 4(A)). The aromatic rings of compound 1 are sandwiched by hydrophobic interactions. For example, residues A28 and Y80 create hydrophobic interactions with the S1 sub-structure. The S1 sub-structure also extends into the sub-pocket of the TAOK1 binding site. Residues I7 and V15 create hydrophobic contacts with the S2 sub-structure, while residue L131 forms a hydrophobic interaction with the phenol at S3 (Figure 4(A)).

The S1 sub-structure of compound 2 forms two hydrogen bonds with hinge residues E79 and C81. The hydrogen bonds with the hinge residues occur with the two amino groups of S1 (Figure 4(B)). Residues I7, V15, A28, Y80, C81, and L131 form a hydrophobic pocket that is occupied by the S1 sub-structure. The S2 and S3 sub-structure of compound 2 are phenols, which facilitate hydrogen bonds with residues H9, K30, and D142 (Figure 4(B)). The hydrophobic benzenoid rings facilitate hydrophobic interactions with residues V15, K30, L131 and A141.

The S1 sub-structure of compound 3 acts as a hydrogen donor to produce two hydrogen bonds with hinge residue C81 (Figure 4(C)). The S1 sub-structure occupies a hydrophobic pocket that consists of residues I7, A28, Y80 and C81 (Figure 4(C)). The sub-structure S2 contains a benzaldehyde that spans a hydrophobic pocket formed by residues V15, A28 and L131. Finally, the nitrobenzene of the S3 sub-structure forms a hydrogen bond with the side chain of residue K30 (Figure 4(C)). Additional hydrophobic interactions occur between residues I7, K30 and A141 and the benzine ring of S3. Together, the interaction analysis suggests that the three identified compounds bind to the active site of TAOK1.

### Common interactions of dual inhibitors

**Figure 5.**
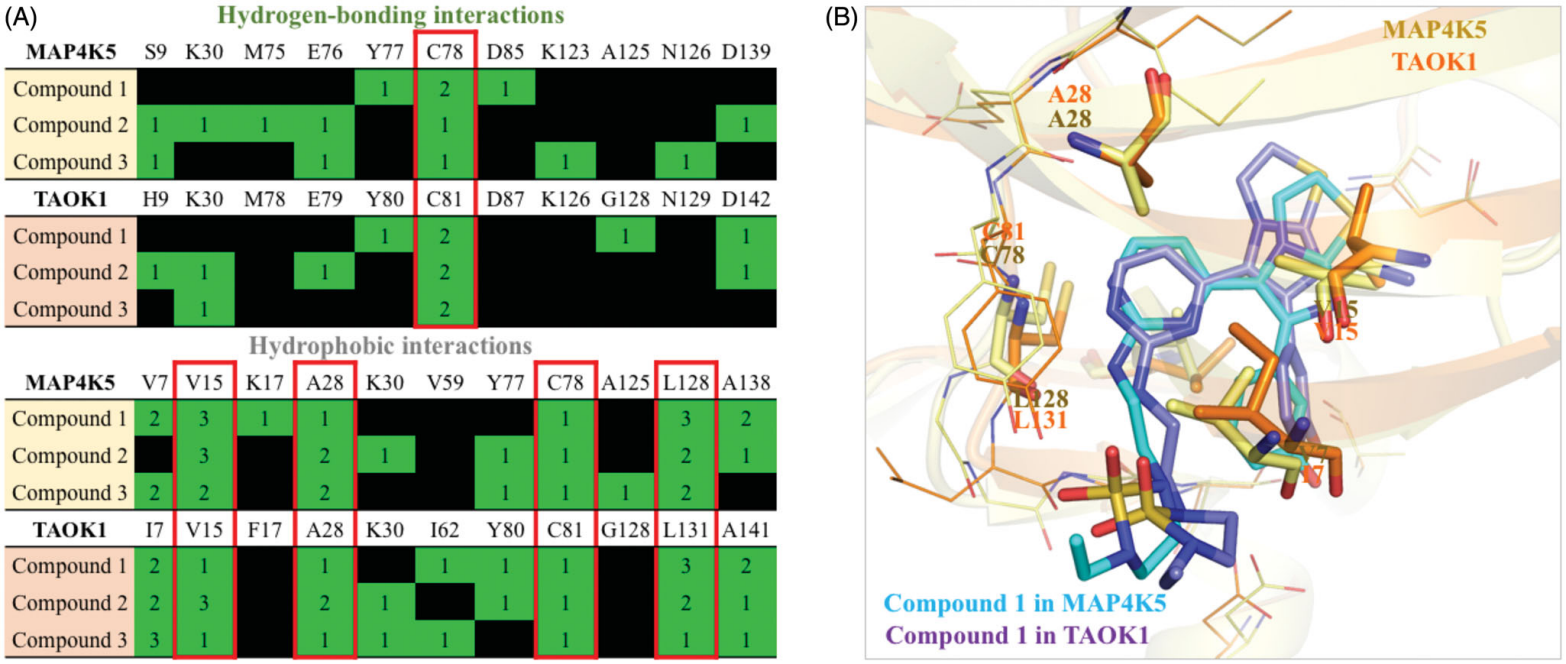
Common interactions of dual inhibitors in MAP4K5 and TAOK1. (A) Hydrogen-bond and hydrophobic interactions for each compound in MAP4K5 and TAOK1 are listed in green. Common interactions between the target kinases is highlighted in red box. (B) The docking pose of compound 1 in MAP4K5 (blue) and TAOK1 (purple) is overlaid. Common interacting residues in target kinase binding site is listed as shown and represented as sticks.

To explore common interactions between the three active compounds, we compared their interactions in both MAP4K5 and TAOK1 binding site. Both MAP4K5 and TAOK1 are grouped in the STE20 kinases^19^^,^^20^. This suggests that the two target kinases may share common characteristics within the ATP-binding site. An analysis revealed a conserved binding site for the MAP4K5 and TAOK1 target proteins (Figure 5(A)). For example, MAP4K5 and TAOK1 have a cysteine hinge residue at positions 78 and 81, respectively, that forms hydrogen bonds with all of the compounds (Figure 5(A)). This suggests that the hydrogen bond with cysteine is an important criterion for inhibition. The identified compounds also form common hydrophobic interactions with four residues. These residues include V15, A28, C78, and L128 of MAP4K5 and V15, A28, C81 and L131 of TAOK1. Interestingly, hydrophobic interactions with these four residues are positioned across various areas in the binding site (Figure 5(B)). These residues may stabilise the compounds within the binding site.

The non-conserved residues do not significantly alter the binding site. For instance, residue V7 of MAP4K5 is substituted with residue I7 in TAOK1 (Figure 5(A)). Both amino acids have small carbon side chains that can form hydrophobic interactions with the inhibitors. Because both of these residues are aliphatic, they have the potential to form similar hydrophobic interactions. For example, compound 1 forms hydrophobic interactions with residues V7 and I7 in MAP4K5 and TAOK1, respectively (Figure 5(B)). Thus, the three identified inhibitors share common interactions between the two target proteins. Together, interactions with these residues can be exploited to create a dual MAP4K5 and TAOK1 inhibitor.

### Compound 1 inhibits multiple cancer cells growth, survival and arrests cell cycle progression

**Figure 6.**
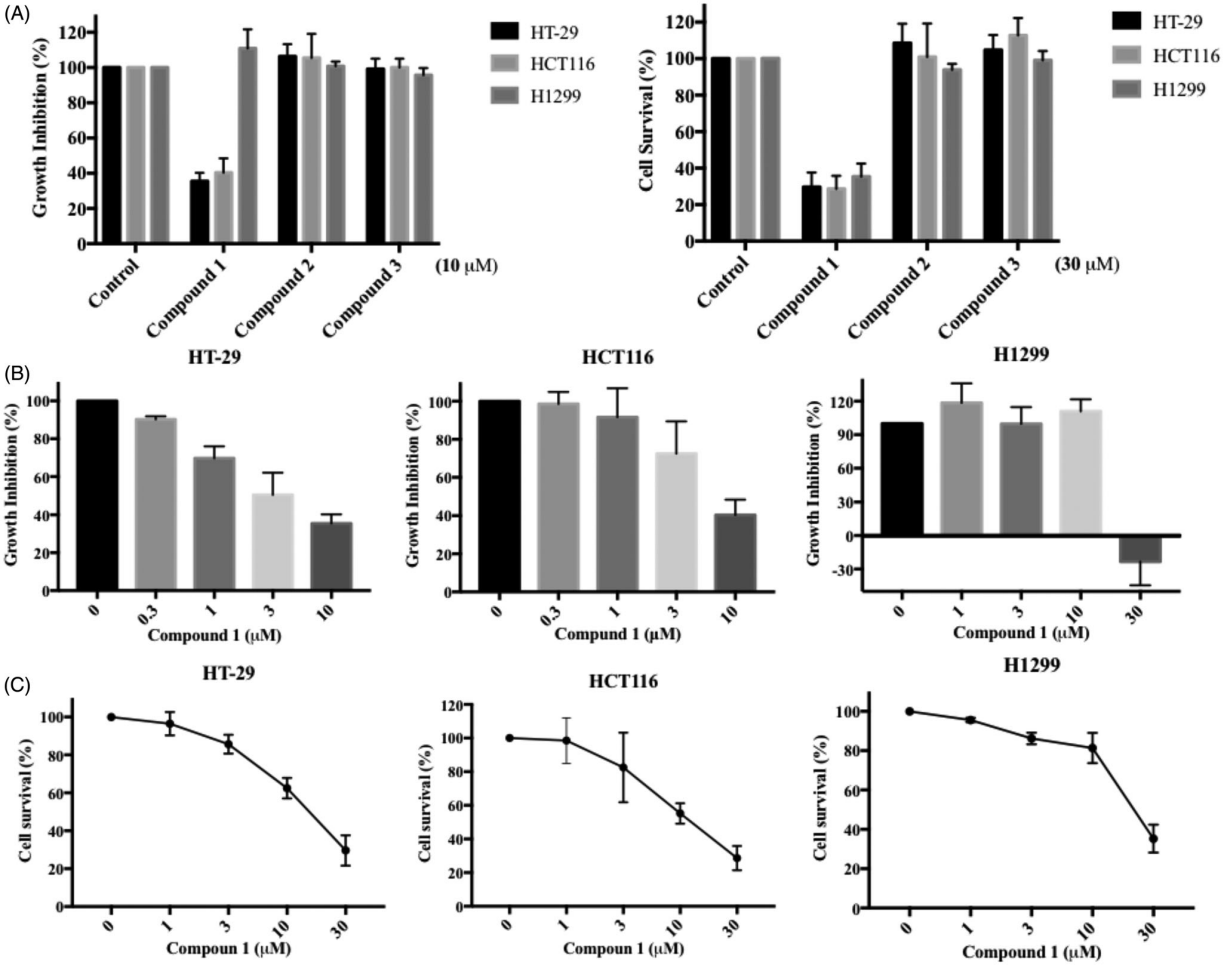
*In vitro* anticancer activity of dual MAP4K5 and TAOK1 inhibitors. (A) The proliferation effect was determined by SRB assay. Colorectal (HT-29 and HCT116) and lung (H1299) cancer cell lines were treated with the indicated compounds at 10 μM for 48 h (left figure). The cell survival was assessed by MTT assay. Three cancer cell lines were treated with the indicated compounds at 30 μM for 72 h (right figure). HT-29, HCT116 and H1299 cancer cell lines were incubated with different concentrations of compound 1 for (B) 48 h to assess anti-proliferative activity and for (C) 72 h to evaluate anti-survival activity. The results are based on at least three independent experiments.

We further evaluated the *in vitro* anticancer impact of the three identified MAP4K5 and TAOK1 inhibitors. Colorectal and non-small cell lung cancer cell lines, HCT116, HT29 and H1299, were used to assess the effect of the compounds on cell proliferation and survival by utilising SRB (Sulforhodamine B) and MTT (3–(4,5-Dimethylthiazol-2-yl)-2,5-diphenyltetrazolium bromide) assays, respectively. Compound 1 significantly inhibited cancer cell proliferation at 10 µM for 48 h and survival at 30 µM for 72 h among the three dual inhibitors tested (Figure 6(A)). This was observed in a concentration-dependent manner. The GI_50_ (50% of growth inhibition) and IC_50_ (50% of maximal inhibitory concentration) for compound 1 in HCT116, HT29 and H1299 cells are 3.9, 6.8, 19.2 µM and 13.3, 13.3, 23.6 µM respectively (Figure 6(B,C)).

**Figure 7.**
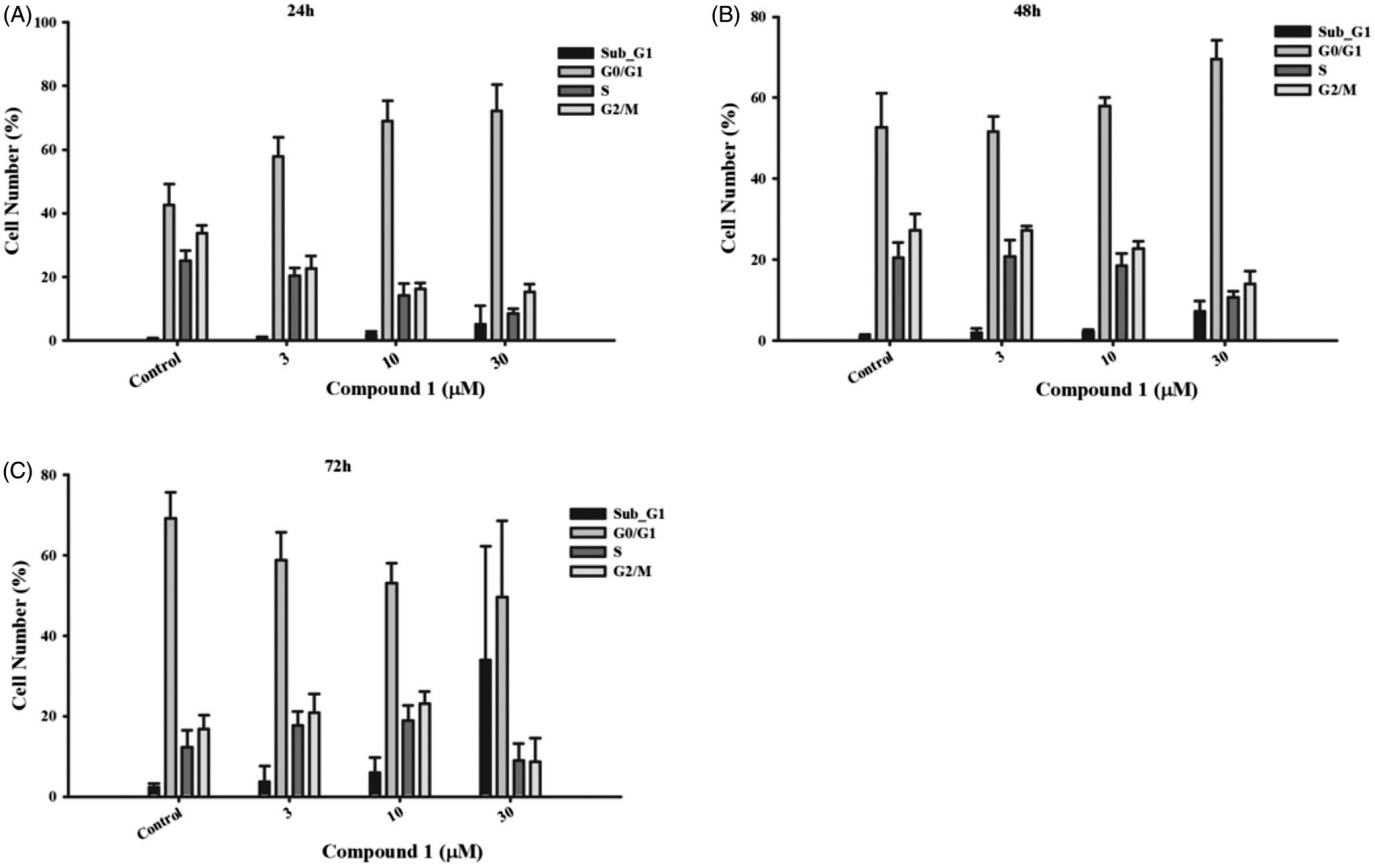
The effect of compound 1 on cell cycle distribution. Colorectal cancer cell line, HT-29, was treated with the indicated concentration of compound 1 for (A) 24 h, (B) 48 h and (C) 72 h to analyse the cell cycle progression, which was determined and performed by utilising BD Accuri™ and C6 Software. The data shown from are at least three independent results.

We next sought to determine how compound 1 would affect the cell cycle of cancer cells using the flow cytometry. After treatment with compound 1 for 24 h in HT29 cells, the number of G0/G1-phase-cells was increased in a concentration-dependent manner (Figure7(A)). As the incubation time progressed, the cell number in the G0/G1 phase decreased concomitantly with the increase in the proportion of cells in the subG1 phase (Figure 7(B,C)). This indicates that compound 1 arrested cancer cells in the G0 phase, thereby evoking apoptosis. Together, this suggests that compound 1 can effectively target cancer cells.

### Selectivity

To evaluate the selectivity of the inhibitors identified in this study, a biochemical assay was conducted by ThermoFisher Scientific. A panel of around 70 kinases from different kinase families as well as all available members of the STE kinase family, was selected for testing (Supplemental Figure 3). The concentration of the compound was selected based on their IC_50_ values. Compound 3 was found to be the most promiscuous of the inhibitors, showing an inhibition of 23 different kinases (Supplemental Figure 3). In comparison, compounds 1 and 2 showed selectivity towards the TKL and STE kinase family, with inhibitory activity against nine and five kinases, respectively. The target kinase in this study, MAP4K5 and TAOK1, belong to the STE20 kinase family. Of the kinases inhibited by compounds 1 and 2, we found eight and three of the kinases to belong to the STE20 kinase family. Together, this implies that the inhibitors identified in this study are selective towards the STE20 kinase family.

### Structure comparison

To better understand the difference between the structures of the identified inhibitors in this study, we compared the structures of the identified inhibitors with FDA-approved kinase drugs that show MAP4K5 or TAOK1 inhibition^40^^,^^41^. Selected drugs exhibited an IC_50_ value of ≤ 10 µM towards MAP4K5 or TAOK1. A similarity matrix was created using the Pearson Correlation Coefficient to produce a similarity score. A similarity score of 0 and 1 denotes little similarity or high similarity, respectively. Overall, the identified inhibitors showed little similarity in structure to the drugs.

For MAP4K5, 13 FDA-approved kinase drugs with inhibitory activity towards MAP4K5 were selected. The structure of compound 1 was closest to Gefitinib, which had a similarity score of 0.48 (Supplemental Figure 4). Axitinib had the highest similarity score of 0.5 when compared to compound 3 (Supplemental Figure 4). For TAOK1, 10 FDA-approved kinase drugs with TAOK1 inhibitory activity were selected. The drugs Sunitinib and Dasatinib had a similarity score of 0.46 and 0.45, respectively, when compared to compound 1. Pazopanib had a high similarity score of 0.48 when compared to compound 3 (Supplemental Figure 5). Overall, no drugs exhibited a similarity score greater than 0.5 when compared to the identified inhibitors. This suggests that the identified inhibitors are novel structures.

We further examined the binding modes between the FDA-approved kinase drugs and the identified inhibitors by comparing their interactions. The drugs were docked to the binding sites of both kinase models. Drugs that successfully generated a docking pose in the binding site were selected for analysis. The compounds were grouped based on their interactions using a hierarchal clustering and Pearson Correlation Coefficient.

When comparing the inhibitors with the MAP4K5 drugs, our analysis found that compound 1 was grouped with Pazopanib. Compound 1 forms hydrogen bonds with residues T77 and D85 as well as hydrophobic interactions to residue K17 and A138. These interactions are not present with Pazopanib (Supplemental Figure 6). Gefitinib was grouped with compound 2. This drug did not form hydrogen-bonding interactions to residues S9, K30 and D139 (Supplemental Figure 6). Finally, compound 3 was grouped with Sorafenib and exhibited hydrogen bonds to residues S9 and E76 (Supplemental Figure 6). This suggests that the identified inhibitors exhibit different interactions when compared to known MAP4K5 inhibitors .

The identified compounds were also grouped with known TAOK1 inhibitors based on their interactions. When comparing compound 1 to Palbociclib, we found hydrogen-bonding interactions to residues Y80 and G128 with the former. These interactions are facilitated by the sulphone and the hydroxyl group of compound 1 (Supplemental Figure 7). Compound 2 is grouped with Imatinib. A hydrogen bond to residue K30 and E79 and a hydrophobic interaction to K30 is present with compound 2, but are not observed with the binding pose of Imatinib (Supplemental Figure 7). Finally, compound 3 was grouped with Pazopanib and forms a hydrogen bond to K30 due to its terminal nitro group. In contrast, pazopanib is unable to form a complementary hydrogen bond to residue K30 in TAOK1. Together, these interactions show a different binding mode between the identified inhibitors and known TAOK1 inhibitors .

## Discussion

In this study, we used a SBVS approach to identify dual MAP4K5/TAOK1 inhibitors. Both kinase targets belong to the STE20 kinase. Members of the STE20 kinase family, such as TAOK1 and MAP4K5, can regulate stress signals that promote cell cycle arrest and apoptosis ^12^. High expression of both TAOK1 and MAP4K has also been implicated in various cancers ^20^^,^^25^. This suggests that an inhibitor targeting multiple kinases in a signalling pathway may have therapeutic benefits.

We selected seven potential inhibitors based on our virtual screening protocol. The compounds were tested in an enzymatic assay at 10 µM, of which compounds 1, 2 and 3 showed inhibitory activity (Figure 2). These compounds exhibit an inhibition of 50% or greater towards the MAP4K5 and TAOK1. In addition, the three compounds produced favourable IC_50_ values of ≤ 10 µM for both targeted kinases (Table 1). Both the MAP4K5 and TAOK1 are grouped within the STE20 kinase family ^19^^,^^20^. As a result, the two targeted kinases may share common interactions with the identified inhibitors. The docking pose of the compounds in MAP4K5 and TAOK1 was further analysed to identify common interactions. We identified key hydrogen bond and hydrophobic interactions within the kinase binding site (Figure 3–4). A formation of a hydrogen bond with the cysteine hinge residue was present in all three inhibitors. Kinase inhibitors typically form one or two hydrogen bonds to the hinge residues^37^^,^^39^. Furthermore, the binding site for both MAP4K5 and TAOK1 is conserved (Figure 5(A)). This suggests that, for the identified inhibitors, a hydrogen bond to C78 (MAP4K5) and C81 (TAOK1) is imperative for MAP4K5 and TAOK1 inhibition. The identified inhibitors contain various ring structures that can be sandwiched by hydrophobic interactions that stabilise them within the binding site. We observed common hydrophobic interactions with four residues that are located near the hinge residues (Figure 5(A)). Interestingly, the compounds produced a similar docking pose in both target kinases, which further suggests the similarity of the binding site between the two related kinases (Figure 5(B)). Residues that are not conserved did not significantly alter the binding site. For example, MAP4K5 contains residue V7, while TAOK1 contains an I7. Both amino acids contain an aliphatic side chain that may facilitate hydrophobic interactions with a heterocyclic structure and the substitution does not significantly alter the binding site. Together, this suggests that the identified inhibitors can bind to the MAP4K5 and TAOK1 binding site for inhibition.

We identified three potential compounds, 1, 2 and 3, from seven candidates via enzyme-based assays. However, compounds, 2 and 3 showed limited effects on cell-based experiments. This may be due to their structure, which may reduce their effectiveness in entering cells. In contrast, compound 1 not only inhibits the growth of colon cancer cells (HCT116 and HT-29), but also lung cancer cells (H1299) (Figure 6(A)). However, a higher dose (30 µM) was needed before inhibitory effects were observed in lung cancer cells. This phenomenon may be due to the low expression level of MAP4K5 protein in lung cancer cell compared with colorectal cancer cell (data from the Human Protein Atlas: http://www.proteinatlas.org).

Both proteins are grouped in the STE20 kinase family, which has been implicated in various diseases ^16^^,^^17^. Studies have suggested that TAOK1 can regulate the MAPK signalling pathways^12^^,^^19^. The MAPK signalling pathway is implicated in many different complex signalling cascades. For example, the Hippo pathway includes TAOK1 and MAP4K5^42^. TAOK1 and MAP4K5 lie upstream within the Hippo pathway and can directly phosphorylate LATS1/2, which in turn activates Yap^43^. In addition to cancer, TAOK1 and MAP4K5 is implicated in neurodegenerative disorders^26–28^. The involvement of TAOK1 and MAP4K5 in the various signalling pathways make them potential therapeutic targets. However, resistance to small-molecule kinase inhibitors has occurred due to mutations within the kinase binding site^7^. Resistance to single target drugs can also arise due to a cancer cell’s adaptive network response, which can recruit and activate other signalling pathways^8^. A multi-targeting kinase inhibitor has the potential to overcome the resistance observed with a single targeting inhibitors.

An induction of cell death in breast cancer and pancreatic cancer cells has been found by inhibiting TAOK1^19^. In our results, compound 1 arrested cancer cells at the G0/G1 phase at 24 h (Figure 7(A)). Previous studies show that TAOK1 can shorten the G1 phase and skip a transient G0-like state to accelerate the speed of the cell cycle^44^. The cells over-accumulated in the G0/G1 phase will be followed by cell growth suppression or enhancement of cell death or apoptosis. It remains to be seen if the inhibitors identified in this study can therapeutically modulate the Hippo pathway. Further study will be needed to determine which context and what roles individual mediators have in inducing the functions of the Hippo pathway. Importantly, the identification of novel small molecule inhibitors could help aid in our understanding of the kinase mediators in the pathway. Our results suggest that a small-molecule targeting TAOK1 and MAP4K5 may provide a beneficial therapeutic option.

Taken together, we used a SBVS approach and identified compound 1 as a dual MAP4K5 and TAOK1 kinase inhibitor. Because signalling pathway of a disease may be complex, we sought to identify a dual kinase inhibitor. Our data suggest that compound 1 can successfully bind to the binding site of both target kinases. This was confirmed through cellular assays, showing favourable IC_50_ levels. A functional assay also revealed that treatment with compound 1 triggers cancer cell inhibition and apoptosis. In total, this suggests that targeting both TAOK1 and MAP4K5 as a novel strategy for therapeutic benefits.

## Author contributions

MWC designed the experiments for bioanalysis and then collected and analysed the data. TEL and KCH performed the *in-silico* studies and analysed the results. MWC and TEL equally contributed to the manuscript. WCHF, CDC, HJT and CRY conducted and supervised the cell-based experiments. LCC, SCY, and TYS analysed the virtual screening data. WJH tested the purity of the compounds. SLP and KCH conceived and supervised the project. All authors have read and agreed to the published version of the manuscript.

## Supplementary Material

Supplemental Material
