# Supplementary material for: Identification of a dual TAOK1 and MAP4K5 inhibitor using a structure-based virtual screening approach
Source: J Enzyme Inhib Med Chem. 2020 Nov 9;36(1):98–108. doi: 10.1080/14756366.2020.1843452 (PMC7655034; doi:10.1080/14756366.2020.1843452)
Supplement: Supplemental Material [file IENZ_A_1843452_SM9050.zip › Sup Fig 3.pdf]

Supplemental Figure 3.

A.

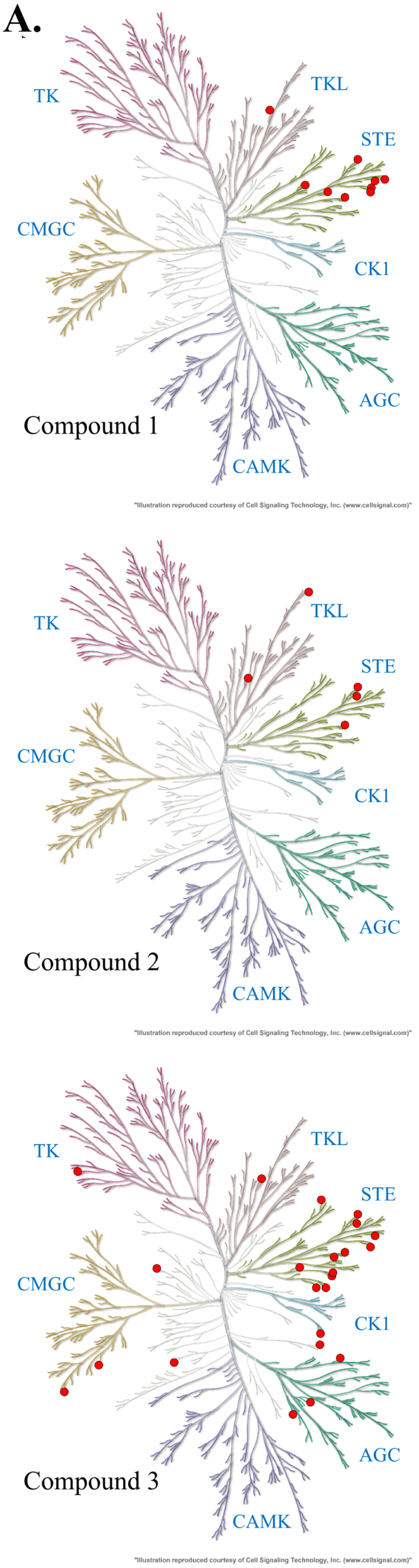

B.

Group	Family	Subfamily	Kinase	Compound 1	Compound 2	Compound 3
AGC	Akt	-	AKT1	1%	21%	13%
AGC	DMPK	ROCK	ROCK2	6%	15%	0%
AGC	GRK	GRK	RHOK	14%	6%	56%
AGC	MAST	MASTL	MASTL	24%	5%	57%
AGC	NDR	-	LATS2	15%	15%	63%
AGC	PDK1	-	PDK1	1%	3%	13%
AGC	PKA	-	PKACa	5%	13%	6%
AGC	PKC	Eta	PKCe	10%	34%	0%
CAMK	CAMK1	-	CaMK1d	5%	1%	8%
CAMK	CAMKL	CHK1	CHK1	10%	0%	14%
CAMK	CAMKL	MELK	MELK	39%	44%	28%
CAMK	DCAMKL	-	DCAMKL1	0%	15%	0%
CAMK	MAPKAPK	MAPKAPK	MAPKAPK2	3%	7%	24%
CAMK	PIM	-	PIM1	14%	3%	22%
CAMK	TSSK	-	TSSK1	10%	12%	7%
CK1	VRK	-	VRK2	46%	45%	79%
CMGC	CDK	CDC2	CDK2	35%	0%	57%
CMGC	CDK	CDK4	CDK4	11%	8%	0%
CMGC	CDK	CDK8	CDK11	16%	29%	40%
CMGC	CDK	CDK9	CDK9	23%	2%	76%
CMGC	CDKL	-	CDKL5	3%	3%	0%
CMGC	MAPK	ERK1	ERK1	45%	7%	0%
CMGC	SRPK	-	MSSK1	14%	0%	2%
Other	Aur	-	AurA	13%	22%	33%
Other	IKK	-	IKKb	13%	18%	46%
Other	IRE	-	IRE1	44%	10%	57%
Other	NEK	NEK2	NEK2	4%	20%	0%
Other	PLK	PLK2	PLK3	15%	20%	72%
Other	ULK	ULK	ULK2	12%	5%	69%
STE	STE7	MEK1	MAP2K1	9%	8%	67%
STE	STE7	MEK1	MAP2K2	20%	8%	61%
STE	STE7	MEK3	MAP2K6	24%	10%	70%
STE	STE7	MEK4	MAP2K4	49%	18%	77%
STE	STE7	MEK5	MAP2K5	10%	0%	53%
STE	STE11	ASK	MAP3K5	19%	0%	43%
STE	STE11	MEKK15	MAP3K19	31%	45%	16%
STE	STE11	MEKK2	MAP3K2	32%	0%	63%
STE	STE20	FRAY	STLK3	51%	8%	34%
STE	STE20	KHS	HPK1 (MAP4K1)	36%	22%	46%
STE	STE20	KHS	GCK (MAP4K2)	19%	53%	76%
STE	STE20	KHS	KHS2 (MAP4K3)	29%	7%	44%
STE	STE20	MSN	MINK	77%	15%	0%
STE	STE20	MSN	TNIK	86%	16%	57%
STE	STE20	MST	MST1	5%	3%	0%
STE	STE20	MST	MST2	0%	2%	0%
STE	STE20	NinaC	MYO3A	51%	12%	37%
STE	STE20	NinaC	MYO3B	57%	42%	52%
STE	STE20	PAKA	PAK1	0%	0%	0%
STE	STE20	PAKA	PAK2	4%	2%	0%
STE	STE20	PAKB	PAK4	2%	6%	28%
STE	STE20	PAKB	PAK6	5%	0%	0%
STE	STE20	SLK	SLK	74%	28%	43%
STE	STE20	TAO	TAO2 (TAOK2)	17%	19%	0%
STE	STE20	TAO	TAO3 (TAOK3)	20%	28%	55%
STE	STE20	YSK	MST3	23%	33%	0%
STE	STE20	YSK	YSK1	6%	12%	0%
STE	STE-Unique	-	COT	22%	4%	23%
STE	STE-Unique	-	NIK	7%	0%	43%
TK	EGFR	-	ErbB2	14%	44%	0%
TK	Eph	-	EphA2	46%	0%	0%
TK	FAK	-	FAK	7%	3%	0%
TK	InsR	-	IGF1R	10%	0%	4%
TK	Syk	-	ZAP70	6%	1%	4%
TK	Tec	-	BTK	13%	17%	70%
TK	Tie	-	TIE2	34%	19%	23%
TKL	IRAK	-	IRAK1	12%	12%	12%
TKL	LISK	TESK	TESK1	26%	31%	55%
TKL	LRRK	-	LRRK2	33%	58%	0%
TKL	MLK	MLK	MLK1	19%	51%	5%
TKL	MLK	ZAK	ZAK	54%	2%	32%
