# Supplementary figures and images for: Identification of a dual TAOK1 and MAP4K5 inhibitor using a structure-based virtual screening approach
Source: J Enzyme Inhib Med Chem. 2020 Nov 9;36(1):98–108. doi: 10.1080/14756366.2020.1843452 (PMC7655034; doi:10.1080/14756366.2020.1843452)

Supplemental Figure 1.

A.

Compound 1

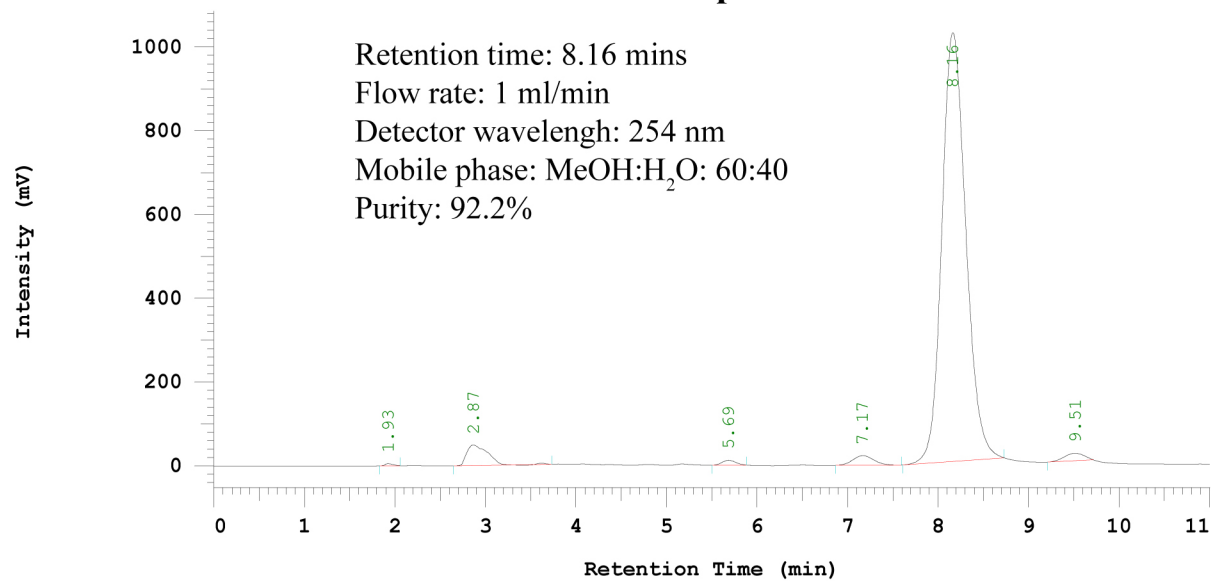

B.

Compound 2

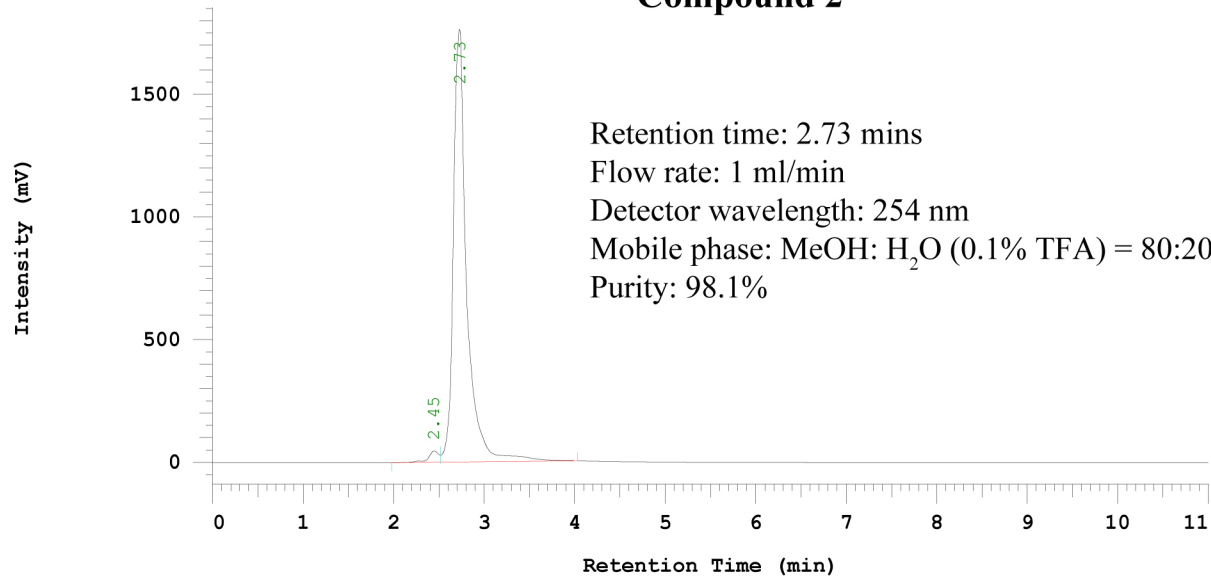

C.

Compound 3

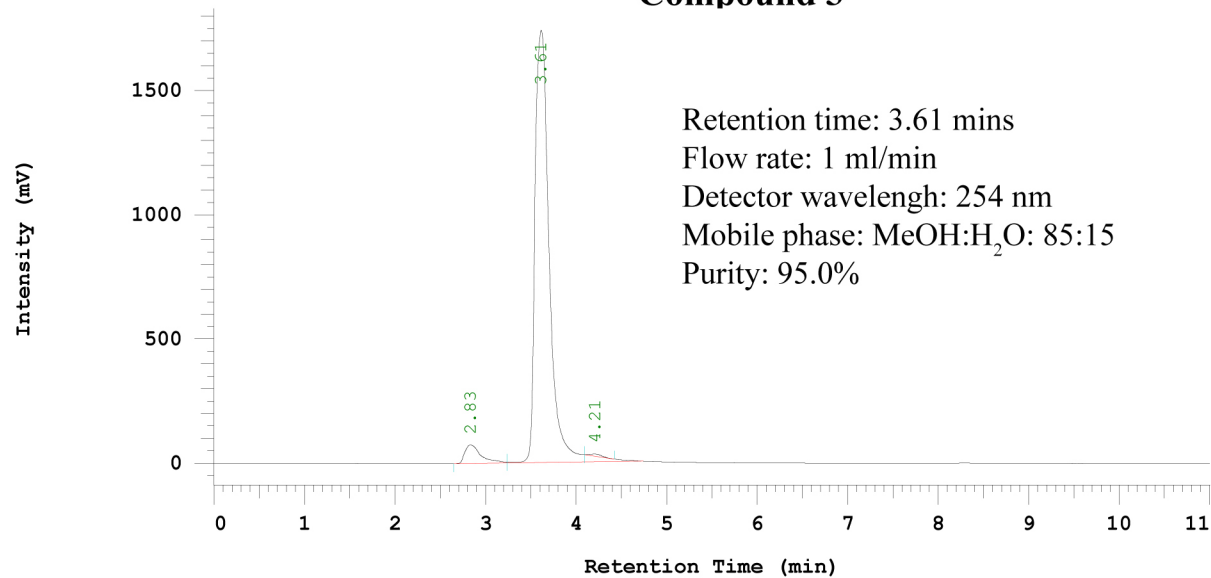

Supplement: Supplemental Material [file IENZ_A_1843452_SM9050.zip › Sup Fig 1.pdf]

Supplemental Figure 2.

A.

MAP4K5

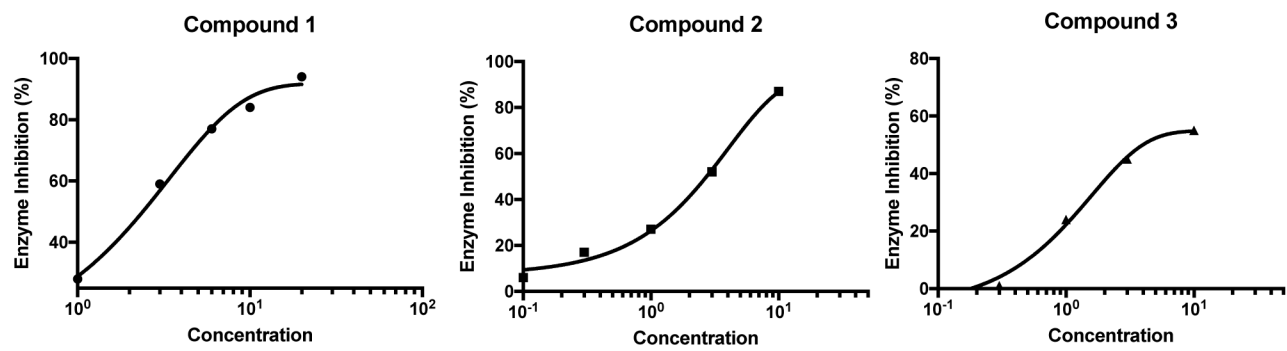

B.

TAOK1

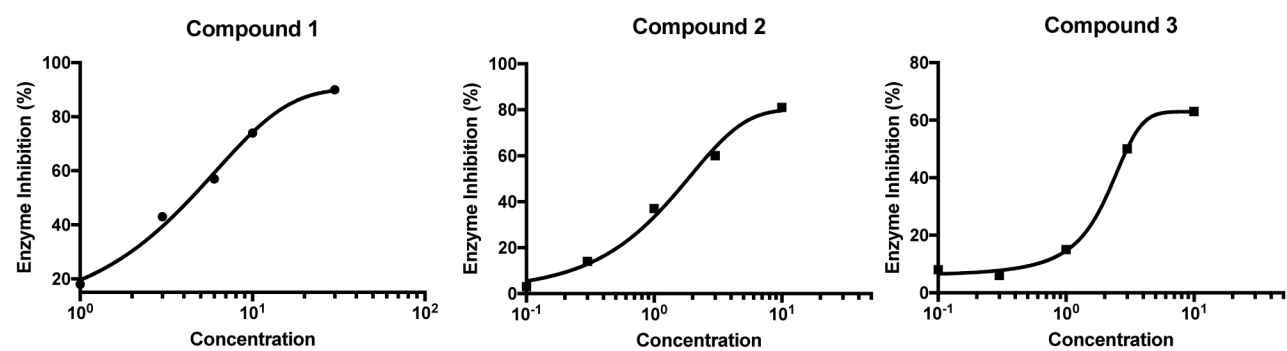

Supplement: Supplemental Material [file IENZ_A_1843452_SM9050.zip › Sup Fig 2.pdf]

**Supplemental Figure 4.**

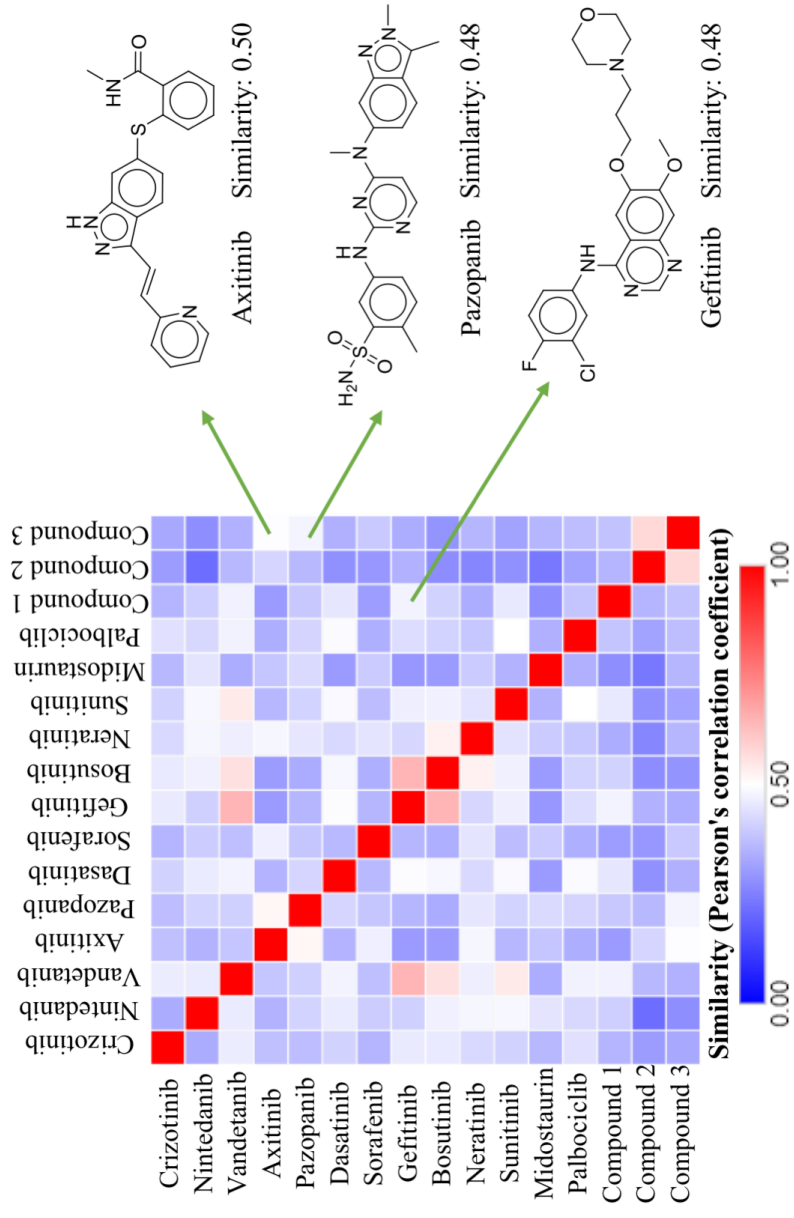

Supplement: Supplemental Material [file IENZ_A_1843452_SM9050.zip › Sup Fig 4.pdf]

## Supplemental Figure 5.

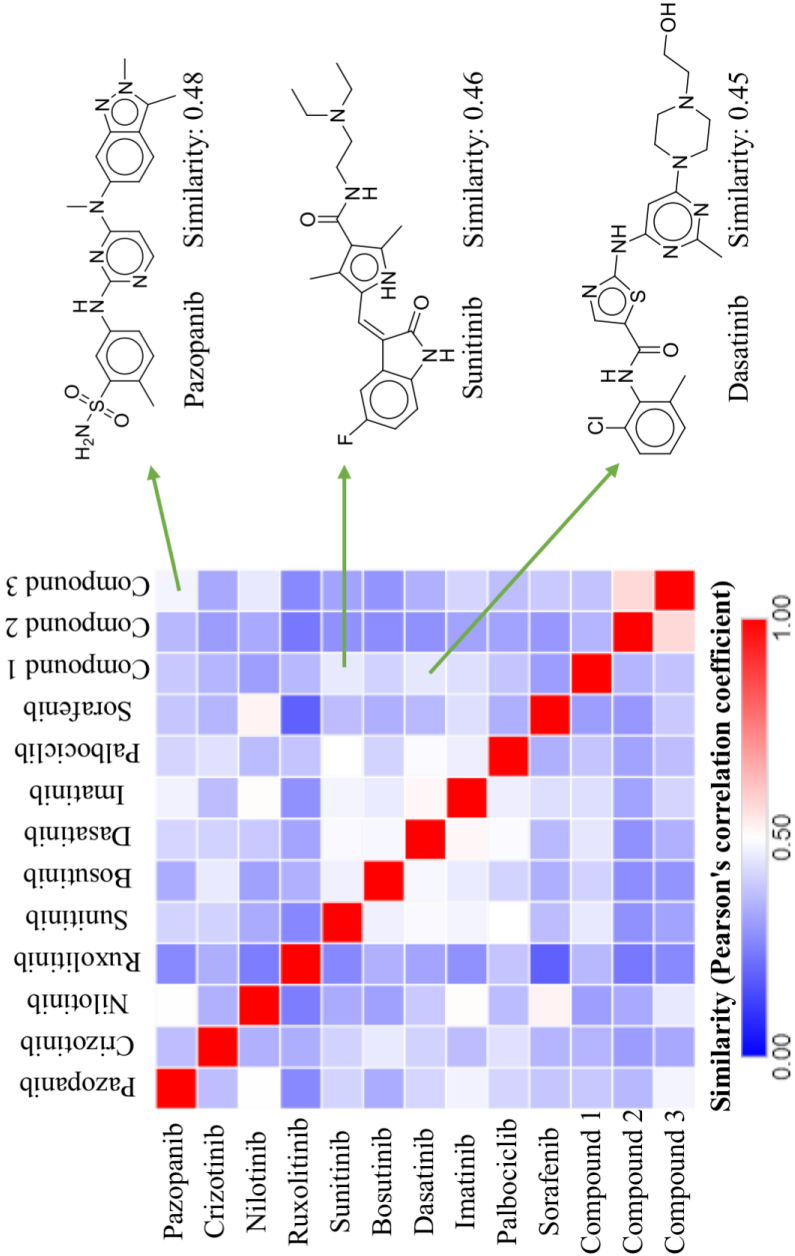

Supplement: Supplemental Material [file IENZ_A_1843452_SM9050.zip › Sup Fig 5.pdf]

Supplemental Figure 6.

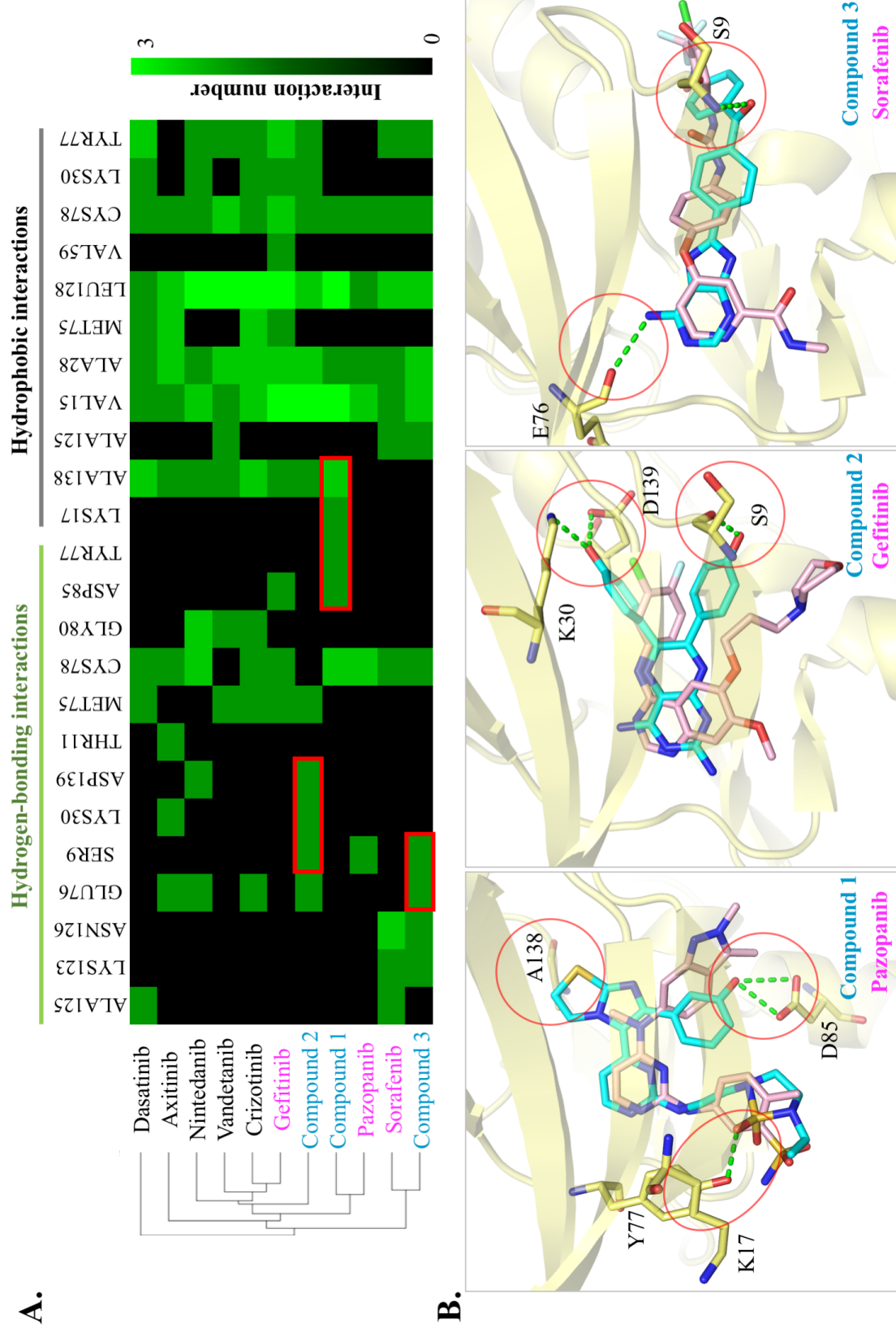

Supplement: Supplemental Material [file IENZ_A_1843452_SM9050.zip › Sup Fig 6.pdf]

# Supplemental Figure 7.

A.

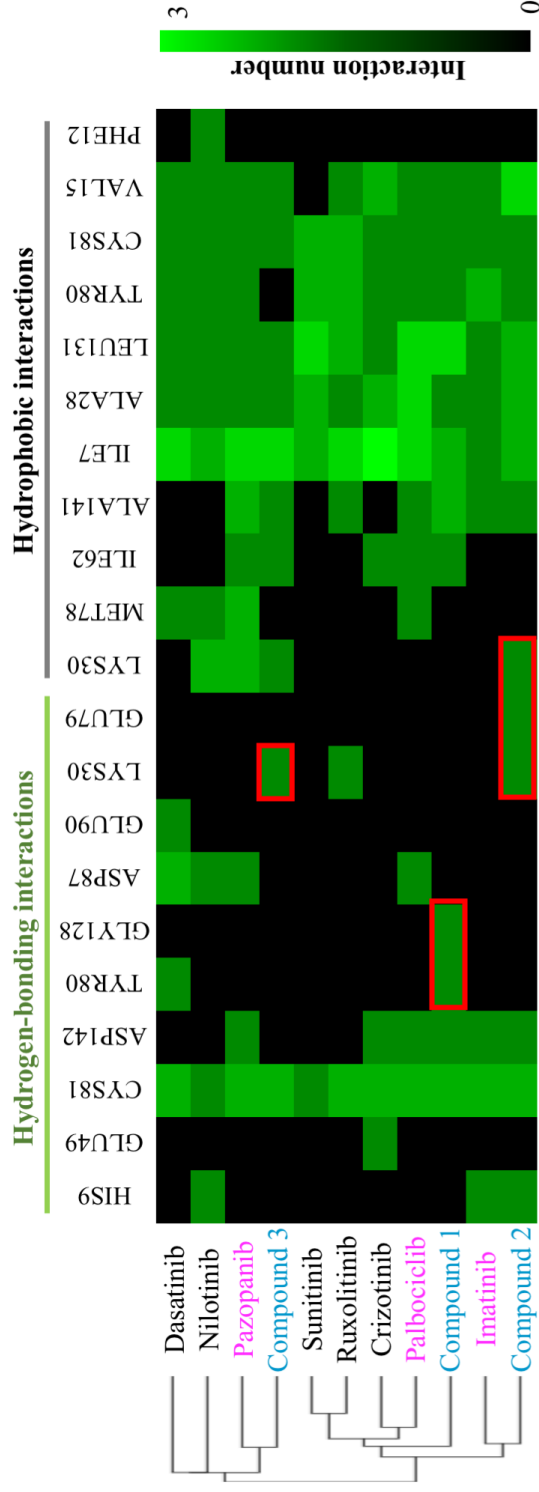

B.

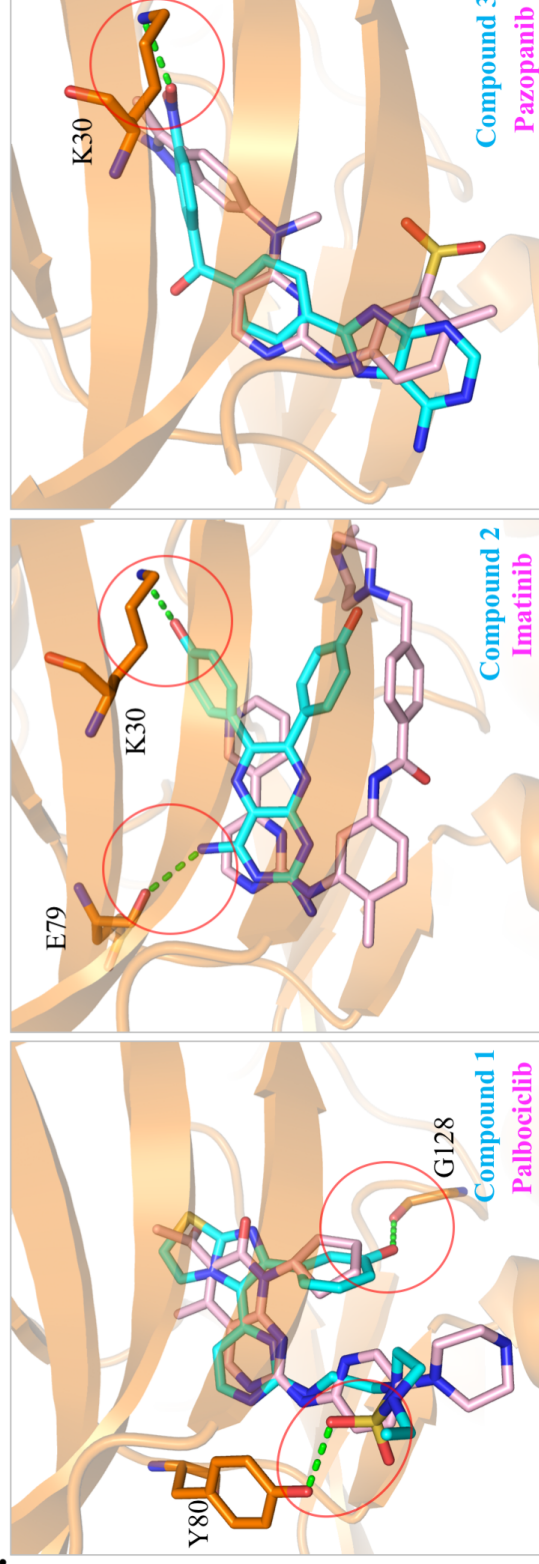

Supplement: Supplemental Material [file IENZ_A_1843452_SM9050.zip › Sup Fig 7.pdf]
